# The Association between Lifestyle Changes and Psychological Distress during COVID-19 Lockdown: The Moderating Role of COVID-Related Stressors

**DOI:** 10.3390/ijerph18189695

**Published:** 2021-09-15

**Authors:** Michal Alon-Tirosh, Dorit Hadar-Shoval, Kfir Asraf, Lubna Tannous-Haddad, Orna Tzischinsky

**Affiliations:** 1 Behavioral Sciences Department, The Max Stern Yezreel Valley College, Yezreel Valley 1930600, Israel; lubnah@yvc.ac.il; 2 Psychology Department, The Max Stern Yezreel Valley College, Yezreel Valley 1930600, Israel; dorith@yvc.ac.il (D.H.-S.); kfira@yvc.ac.il (K.A.); 3 Educational Counseling Department, The Max Stern Yezreel Valley College, Yezreel Valley 1930600, Israel; orna@yvc.ac.il

**Keywords:** health behavior, depression, anxiety, stress, cigarette smoking, alcohol consumption, physical activity, sleep quality, eating habits, nature of change

## Abstract

The COVID-19 pandemic and the accompanying circumstances (lockdown and social distancing) have been found to cause lifestyle habit changes. While negative changes (adopting risky behaviors) are known to be associated with high psychological distress, the effect of positive changes (adopting health-enhancing behaviors) has yet to be investigated. This study examined the association between the nature of changes and psychological distress, in addition to the moderating effect of “COVID-related stressors”. Online questionnaires, completed by 1969 participants, assessed the following: lifestyle changes due to COVID-19 with regard to cigarette smoking, alcohol consumption, physical activity, sleep quality, and eating habits; COVID-related stressors; Depression, Anxiety, and Stress Scale. Positive and negative changes were associated with higher psychological distress compared to no lifestyle change, and there was a moderating effect of COVID-related stressors in the association between COVID-related lifestyle changes and distress. In conclusion, to reduce psychological distress in overwhelming circumstances such as the COVID-19 pandemic, people should carefully consider whether to make changes in their lifestyle habits before doing so, even if these changes are perceived as positive and health-enhancing. Furthermore, efforts should be focused on reducing the perception of stress from COVID-19 by working on personal and mental perceptions of the situation.

## 1. Introduction

The COVID-19 pandemic has been shown to have multidimensional effects, endangering not just health, but also economic stability, civil rights, and mental health [1]. On the psychological dimension, the feelings of uncertainty, ambiguity, and loss of control caused by the pandemic and the resultant conditions (such as quarantine and lockdowns, social distancing, and occupational changes) have been shown to be strongly associated with psychological distress [2,3]. Furthermore, the pandemic and the accompanying circumstances have been found to be associated with high levels of anxiety [3,4,5], depression [6,7], stress [8], and stress disorder [9].

Lockdowns in particular are associated with psychological distress, as they lead to social isolation, loneliness, and lifestyle disruptions and changes. Studies have indicated that the circumstances accompanying the COVID-19 pandemic resulted in massive lifestyle changes, as reflected in health behavioral changes [10]. For example, Spanish adults reported changes in physical activity, alcohol use, fruit and vegetable consumption, smoking, and sleep in the first three weeks of confinement due to COVID-19 [11]. A study conducted in Italy reported changes in lifestyle behaviors, such as a change in diet, decreased smoking, more sleep, and increased frequency of physical training [12]. Arora and Grey [10] reviewed evidence that alcohol consumption increased during the early stages of the pandemic in Australia, the United Kingdom, and the United States. Another study [13] used data from an application that tracked users’ physical activity to document the decline in daily steps among users in 10 countries. Additionally, interviews among 2250 adults in the UK, revealed that 35% reported eating more food or less healthy food than normal, and 19% reported increased alcohol use than normal [14].

These changes in lifestyle habits and health behaviors can in turn affect a person’s psychological state. For example, Stanton et al. found that adults who reported negative changes in physical activity, sleep, alcohol intake, and cigarette smoking demonstrated higher psychological distress, which was expressed in terms of more severe depression, anxiety, and stress symptoms [15]. This finding led the authors to recommend that the public be encouraged to make behavioral changes related to adopting a healthier lifestyle. This recommendation is likely based on the assumption that the nature of the behavioral changes influences the psychological reaction; that is, if negative lifestyle changes are associated with increased psychological distress, positive lifestyle changes should decrease psychological distress. Yet, the association between positive lifestyle changes and psychological distress was not examined by Stanton et al. [15]. Furthermore, findings regarding the effect of changes resulting from various life events on one’s psychological state have indicated that the nature of the change does not matter, and that positive changes have the same impact on psychological well-being as do negative changes [16,17,18]. Therefore, the assumption that adults who will adopt a healthier lifestyle during the COVID-19 pandemic will demonstrate decreased psychological distress has yet to be examined.

Therefore, the current study examines how health-enhancing changes (henceforth, “positive” lifestyle changes) during lockdown due to COVID-19 are associated with psychological distress. The study focuses on changes in five behaviors associated with health: cigarette smoking, alcohol consumption, physical activity, sleep quality, and eating habits. We hypothesized that there is a difference in psychological distress between people who experienced changes in lifestyle due to COVID-19 (both positive and negative lifestyle changes) and people who did not experience such changes at all.

In addition to the nature of the behavioral changes, its magnitude (that is, the number of changes people make at the same time and their relative size) is also associated with psychological distress—the stronger the change, the greater the distress. Since the present study was conducted in the context of the COVID-19 pandemic, “COVID-related stressors” could be a moderating factor in the expected association between the magnitude of lifestyle changes due to COVID-19 and psychological distress. Evidence from a range of countries and cultures has suggested that adults have experienced multiple COVID-related stressors, including financial and health-related stress, social isolation, and difficulty in obtaining needed supplies, as well as persistent worries about one’s self or family members becoming ill [19,20,21]. Furthermore, an association between people’s experience of multiple COVID-related stressors and psychological distress symptoms has been reported [19]. In a large sample of US adults with no reported history of mental health problems, more than 25% reported psychological distress in the early weeks of the pandemic (March, 2020) [22]. Similarly, in a sample of American and Israeli participants, “COVID-19-related stressors” were significantly associated with anxiety and depression [2]. 

It is important to note that the experience of COVID-related stressors is subjective. During crises such as the COVID-19 pandemic, individuals exhibit diverse characteristics and response styles that determine their stress level [23,24]. For example, some of the participants in a recent study reported no COVID- related stressors at all [2].

**Figure 1:**
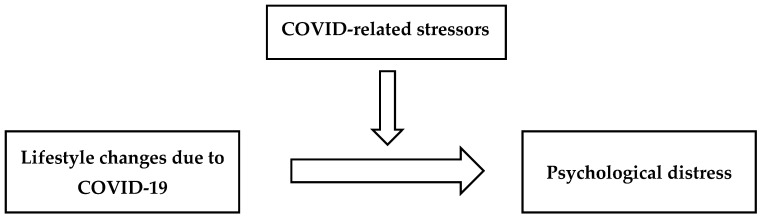
Theoretical model of COVID-related stressors as a moderating factor on the association between lifestyle changes due to COVID-19 and psychological distress.

In light of the above findings, our second research hypothesis was that the level of COVID-related stressors moderates the association between lifestyle changes due to COVID-19 and psychological distress, whereby there is a stronger association in those who perceive the event as more stressful (Figure 1).

## 2. Materials and Methods

### 2.1. Participants and Data Collection

The current study used data collected by the authors in a cross-sectional survey administered in Israel. The survey was designed to assess the public’s demographic characteristics and their immediate psychological and behavioral responses during the COVID-19 pandemic via an anonymous online questionnaire using Qualtrics (accessed on 15 June 2021 https://www.qualtrics.com). The survey was sent to participants online by iPanel (accessed on 15 June 2021 https://www.ipanel.co.il), a large Israeli panel service. iPanel has reported having the largest online access panel in Israel, with over 100,000 active panel members aged 12 years and more. Inclusion criteria defined to iPanel for the survey included being between 18 and 75 years of age and speaking the language in which the survey was administered (either Hebrew or Arabic). No exclusion criteria were applied. Questionnaire completion was voluntary, and respondents were told that they could stop their participation at any point. Participants who completed the survey were excluded from the final analysis if they failed attention checks, if they completed the measures in less than 10 minutes, and if their responses were implausible (e.g., they chose the same answer throughout the questionnaire). The complete study protocol was approved by the College Institutional Review Board (2020-54 YVC EMEK).

The final analysis included 1969 participants. More than half of the sample (55.1%) was female. Participants’ mean age was 40.4 (SD = 13.76) years, with a mean years of education of 14.62 (SD = 2.50) years. Most participants lived in urban areas (75.06%), and about two-thirds of the participants were married (*n* = 1238). Regarding the economic influence of COVID-19, about two-thirds of the participants had their income impacted in some way. Participants’ demographic data are presented in Table 1.

### 2.2. Measures

#### 2.2.1. Demographic Questionnaire

This questionnaire consisted of questions on age, years of education, gender, income status, and work status since the onset of the COVID-19 pandemic.

#### 2.2.2. Lifestyle Changes due to COVID-19

Lifestyle changes due to COVID-19 were measured using two questions (on the nature and magnitude of change) for each of the five lifestyle habits, as follows:

Nature of change: Participants were asked to indicate whether they changed their behavior with regard to each of the five lifestyle habits and to refer to the nature of the change (no change/ positive change/ negative change). A similar methodology has been used in previous studies [15,25,26].

Cigarette smoking: Participants were asked to answer the following question: “Since the outbreak of COVID-19 and the beginning of the lockdown and quarantine, did you change your cigarette smoking habits?”. Participants were presented with three possible answers: A. I reduced the number of cigarettes (positive change); B. I increased the number of cigarettes (negative change); C. I did not change the number of cigarettes (no change).

Alcohol consumption: Participants were asked to answer the following question: “Since the outbreak of COVID-19 and the beginning of the lockdown and quarantine, did you change your alcohol consumption habits?”. Participants were presented with three possible answers: A. I reduced my alcohol consumption (positive change); B. I increased my alcohol consumption (negative change); C. I did not change my alcohol consumption (no change).

Physical activity: Participants were asked to answer the following question: “Since the outbreak of COVID-19 and the beginning of the lockdown and quarantine, did you change your physical activity habits?”. Participants were presented with three possible answers: A. I increased my physical activity (positive change); B. I reduced my physical activity (negative change); C. I did not change my physical activity (no change).

Sleep quality: Participants were asked to answer the following question: “Since the outbreak of COVID-19 and the beginning of the lockdown and quarantine, did your sleep quality change?”. Participants were presented with three possible answers: A. My sleep quality improved (positive change); B. My sleep quality deteriorated (negative change); C. My sleep quality did not change (no change).

Eating habits: Participants were asked to answer the following question: “Since the outbreak of COVID-19 and the beginning of the lockdown and quarantine, did your eating habits change?”. Participants were presented with three possible answers: eating habits improved A. My eating habits have improved and I began eating healthier food (positive change); B. My eating habits deteriorated and I began eating more unhealthy food (negative change); C. I did not change my eating habits (no change).

The magnitude of change: Participants were asked to estimate the magnitude of change on a scale of 0–2, where 0 indicates no change, 1 indicates moderate change, and 2 indicates substantial change. The magnitude of change score was the sum of the scores for each of the lifestyle change variables and ranged from 0–10.

#### 2.2.3. COVID-Related Stressors

Participants rated how stressful each of the 13 COVID-related stressors had been since the beginning of the lockdown, ranging from 1 (not at all stressful) to 4 (very stressful) [2,19,27]. Stressors included financial problems, inability to spend time with friends or family, changes to normal routines, cancellation of travel plans, challenges at home, trouble obtaining needed supplies or services, hearing distressing news reports, uncertainty about self or others getting COVID-19, difficulty completing work or educational responsibilities, increased work or family responsibilities, and uncertainty about the future. The total COVID-19-related stressors score was computed for each participant by summing the scores for all 13 items; higher scores indicate greater stress. Internal consistency (Cronbach’s alpha) was 0.87.

#### 2.2.4. Depression, Anxiety, and Stress Scale

We used the Hebrew version of the Depression, Anxiety, and Stress Scale (DASS-21) scale (retrieved from the DASS21 website 18 June 2021 http://www2.psy.unsw.edu.au/dass/), which was originally developed in 1995 [28]. It evaluates the total score of 21 items, comprising depression (7 items), anxiety (7 items), and stress (7 items) dimensions. All items are rated using a 4-point Likert scale, ranging from never (0) to most of the time (3). For the depression scale, a score above 11 indicates severe depression; for the anxiety scale, a score above 8 indicates severe anxiety; and for the stress scale, a score above 9 indicates moderate or severe stress. The DASS-21 demonstrates a good reliability and validity [29] in clinical and non-clinical samples. In the current study, the internal reliability of this questionnaire (Cronbach’s alpha) was 0.96 for the total score, 0.91 for depression, 0.90 for anxiety, and 0.91 for stress.

### 2.3. Statistical Analysis

All analyses were conducted using IBM SPSS version 26. Demographic data were analyzed using independent-samples t-tests or Pearson’s chi-squared tests. None of the primary outcome measures violated the normality assumption, as the skewness and kurtosis were between +2 and −2. Differences in distress in each of the five lifestyle change variables were examined using Welch’s one-way ANOVA, together with Games–Howell post hoc tests. The Games–Howell test does not assume equal sample size or homogeneity of variances. Bootstrapped confidence intervals (95% bias-corrected and accelerated [BCa] CIs, 5000 samples) are reported for pairwise comparisons, where the absence of zero in the CI indicates statistical significance. Moderation analysis was performed using Model 1 of Hayes’ PROCESS macro (v 3.5) [30] for SPSS. The predictors were COVID-related stressors and the magnitude of change, with age, gender, and years of education as covariates. The dependent variable was distress (DASS total score). The continuous variables were standardized (Z-scored) before being entered into the model. Bootstrap-derived CIs are reported for the coefficients. Effect size estimators were Eta-squared (η2) for the ANOVA, Hedges’ g for Games–Howell tests, and R^2^ for multiple regression.

## 3. Results

### 3.1. Cohort Description of the Main Research Variables

Table 2 reports the descriptive statistics of the cohort’s main study variables. While the mean and median scores of the three DASS-21 subscales were within the range considered as “normal”, several participants scored outside the range. More than one-third of participants (35.96%, *n* = 705) reported depression (a score higher than 4), a quarter of the participants (25.76%, *n* = 505) reported anxiety (a score higher than 3), and a quarter of the participants (25.86%, *n* = 507) reported stress (a score higher than 7). Henceforth, we use the DASS total score to indicate the level of psychological distress to examine the combined effect of these three psychological distress variables. Regarding the nature of change, 51.48% of participants reported no change in their alcohol consumption, while 24.7% and 23.36% reported positive and negative changes, respectively. Regarding cigarette smoking, 82.03% of participants reported no change, 7.52% reported a positive change, and 10.44% reported a negative change. Regarding eating habits, 21.81% of participants reported no change, while 43.63% and 34.55% reported positive and negative changes, respectively. Regarding sleep quality, 29.94% of participants reported no change, 62.87% reported a positive change, and 7.17% reported a negative change. Regarding physical activity, 23.48% of participants reported no change, 21.96% reported a positive change, and 54.54% reported a negative change.

The mean magnitude of change was 3.24 (SD = 1.70), and the median was 3 (range 0–10), which indicates that more than half of the participants experienced a change in more than two lifestyle habits, at different magnitudes. The mean of COVID-related stressors was 32.86 (SD = 8.22), and the median was 33 (range 13–52).

### 3.2. The Association between the Nature of Lifestyle Change Due to COVID-19 and Psychological Distress

The first hypothesis surmised that there will be a difference in psychological distress between people who experienced positive or negative changes in lifestyle due to COVID-19 and people who did not experience such changes. To examine this hypothesis, we conducted Welch’s one-way ANOVA of the five lifestyle variables (alcohol consumption, cigarette smoking, eating habits, sleep quality, and physical activity). The mean and standard deviations are presented in Table 3.

Although the groups of the various lifestyle variables differed in some demographic measures, such as age, gender, and years of education, including these variables as covariates did not meaningfully alter the amount of variance uniquely explained by the change in lifestyle. Therefore, we present the parsimonious models, with change as the sole variable.

*Alcohol consumption*: The ANOVA was statistically significant [*F*(2283.73) = 33.97, *p* < 0.001, η^2^ = 0.096] with a medium effect size, and post-hoc analyses showed that the group with no change in alcohol consumption had significantly less distress than the group with the positive change [*p <* 0.001, CI = −6.91, −3.36, Hedges’ g = 0.61] and the negative change [*p <* 0.001, CI = −8.22, −4.60, Hedges’ g = 0.77]. The positive and negative groups were not statistically different from each other [*p =* 0.508, CI = −4.11, 1.47, Hedges’ g = 0.12].

*Cigarette smoking*: The ANOVA was statistically significant [*F*(2, 260.73) = 12.04, *p* < 0.001, η^2^ = 0.017] with a small effect size, and post-hoc analyses showed that the group with no change in cigarette smoking experienced significantly less distress than the group with the negative change [*p <* 0.001, CI = −7.95, −3.34, Hedges’ g = 0.42], but was not different from the group with the positive change [*p =* 0.902, CI = −2.97, 1.91, Hedges’ g = 0.04]. The group with the negative changes exhibited significantly higher distress than the group with the positive change [*p =* 0.004, CI = 1.93, 8.18, Hedges’ g = 0.33].

*Eating habits*: The ANOVA was statistically significant [*F*(2, 1216.64) = 126.15, *p* < 0.001, η^2^ = 0.065] with a medium effect size, and post-hoc analyses showed that the group with no change in eating habits showed significantly less distress than the group with the positive change [*p <* 0.001, CI = −6.80, −4.89, Hedges’ g = 0.57] and the negative change [*p <* 0.001, CI = −8.41, −6.37, Hedges’ g = 0.77]. The group with the positive change exhibited significantly less distress than the group with the negative change [*p =* 0.028, CI = −2.72, −0.41, Hedges’ g = 0.13].

*Sleep quality*: The ANOVA was statistically significant [*F*(2, 277.03) = 360.85, *p* < 0.001, η^2^ = 0.273] with a large effect size, and post-hoc analyses showed that the group with no change in sleep quality exhibited significantly less distress than the group with the positive change [*p <* 0.001, CI = −10.69, −8.98, Hedges’ g = 1.00] and the negative change [*p <* 0.001, CI = −28.1, −21.38, Hedges’ g = 2.83]. The group with the positive change had significantly less distress than the group with the negative change [*p <* 0.001, CI = −18.29, −11.44, Hedges’ g = 1.21].

*Physical activity*: The ANOVA was statistically significant [*F*(2, 669.66) = 18.29, *p* < 0.001, η^2^ = 0.024] with a small effect size, and post-hoc analyses showed that the group with no change in physical activity exhibited significantly less distress than the group with the positive change [*p =* 0.003, CI = −4.35, −1.10, Hedges’ g = 0.26] and the negative change [*p <* 0.001, CI = −5.82, −2.99, Hedges’ g = 0.37]. The group with the positive change exhibited significantly less distress than the group with the negative change [*p =* 0.073, CI = 0.16, 3.17, Hedges’ g = 0.14; while the *p*-value was above 0.05, the bootstrap-derived CI does not include zero, which suggests that the comparison is statistically significant].

These results suggest that both positive and negative changes are associated with more psychological distress compared to no lifestyle change. Additionally, in most of the lifestyle measures, a negative change in lifestyle was associated with more distress than a positive change.

### 3.3. The Moderating Effect of COVID-Related Stressors on the Association between the Magnitude of Change and Psychological Distress

The second hypothesis was that the level of COVID-related stressors moderates the association between lifestyle changes due to COVID-19 and psychological distress, such that among those who perceive the event as more stressful, there will be a stronger association. To test this hypothesis, we first applied Pearson’s correlations (bootstrapped 95% BCa CI, 5000 samples) to examine whether the magnitude of change was associated with more distress. We found a statistically significant and positive correlation [r = 0.36, *p* < 0.001, CI = 0.32, 0.40], which suggests that more change, regardless of its nature, is associated with higher distress.

We then examined whether COVID-related stressors moderate this aforementioned association. As the distress measure (the DASS total score) includes the stress scale of the DASS questionnaire, we examined the correlation between COVID-related stressors and the DASS total score. The two variables were significantly positively correlated [r = 0.47, *p* < 0.001, CI = 0.43, 0.50], which suggests that the two measures were only moderately associated.

**Figure 2:**
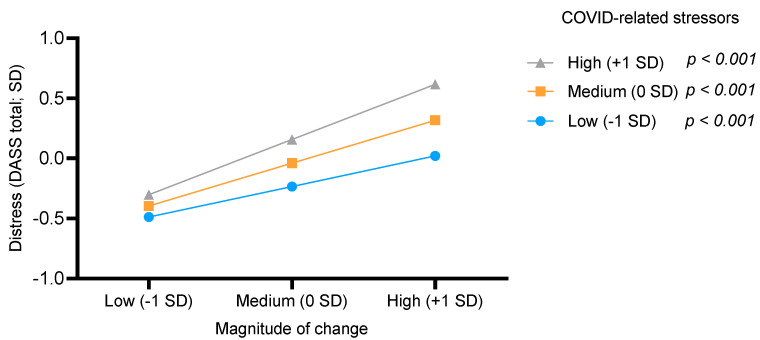
COVID-19 related stress moderated the association between the magnitude of change and psychological distress.

The model of the moderation analysis included age, gender, income status since the COVID-19 pandemic, and work status during the pandemic as covariates, as these variables were significantly correlated with the DASS total score. Years of education was weakly correlated with the DASS total score, and therefore was omitted from the model. The model was significant [*F*(81, 938) = 106.06, *p* < 0.001] and explained 30.45% of the variance (R^2^). The addition of COVID-related stressors X magnitude of change interaction term [b = 0.10, *p* < 0.001, CI = 0.05, 0.14] was significant [*F*(11, 938) = 29.73, *p* < 0.001] and added 1.07% to the explained variance. Probing the interaction, we examined the change in the distress score as a function of COVID-related stressors X magnitude of the change interaction term. Both the independent variables were split into three conditions: low (−1 SD), medium (0 SD), and high (+1 SD). The slopes of the three conditions of the moderator (COVID-related stressors) were statistically significant, and hence, the associations between distress and magnitude of change in the low [b = 0.09, t = 3.31, *p* < 0.001], medium [b = 0.19, t = 9.48, *p* < 0.001], and high [b = 0.29, t = 10.71, *p* < 0.001] categories were all different from zero. The trajectory was similar for the three conditions; i.e., the higher the magnitude of change, the greater the distress. However, this effect was the strongest when the COVID-related stressors score was also high (Figure 2).

## 4. Discussion

The COVID-19 pandemic has impacted all areas of life globally. Along with increased infection and mortality rates, the pandemic has also caused psychological distress. In the current study, more than one-third of participants were found to suffer from depression, and about a quarter of the participants reported suffering from anxiety and/or stress. These findings are in line with existing research on psychological distress related to COVID-19 [4,8,31,32,33]. The pandemic has also had a significant impact on people’s everyday life, causing lifestyle changes [11,12,15]. In the current study, more than half of the participants experienced a change in more than two lifestyle habits, such as cigarette smoking, alcohol consumption, physical activity, sleep quality, and eating habits.

The goal of the current study was to examine the association between lifestyle changes due to COVID-19 and psychological distress. The study findings support the first hypothesis that psychological distress differs between people who experienced positive or negative changes in lifestyle due to COVID-19 and people who did not experience such changes. The results demonstrate that people who experienced lifestyle changes due to COVID-19 exhibited more psychological distress than people who did not experience such changes. Although these findings indicate that the nature of change (whether it is positive or negative) makes some difference (people who experienced negative lifestyle changes demonstrated more psychological distress than those who experienced positive changes), all changes were associated with high psychological distress. These findings are in line with what is already known about the association between stress and life events [16,17], whereby all life events that include changes, even if they are perceived as positive events, are associated with stress and distress. The findings also support those of animal studies that have demonstrated an association between stress and changes. Examples include manipulation that causes stress in rodents that are changing something in their routine, such as changing of food [34], and change in the amount of bedding in the cage [35].

The findings that positive changes are also associated with high psychological distress during the COVID-19 pandemic are important when considering health-enhancing behaviors. While some experts recommend adopting health-enhancing behaviors to reduce psychological distress [15,26,36], the current study argues otherwise, as it highlights the association between positive changes (such as health-enhancing behaviors) and distress. For example, the study demonstrates that some participants reduced their alcohol consumption, a finding that could be explained by alcohol consumption usually being a social activity in Israel, which occurs in pubs and restaurants (and these were closed during the lockdown and quarantine).These study participants who reduced their alcohol consumption (which is considered a health-enhancing behavior) reported more psychological distress than people who maintained their drinking habits. It is important to note that the current study does not attend to the health-enhancing behaviors’ possible association with physiological health, as its focus is on psychological distress. This study also does not rule out the possible long-term benefits of these positive lifestyle changes on psychological distress. However, it does indicate that in the immediate context of the pandemic and lockdown, any change, positive or negative, is associated with greater psychological distress.

The findings also support the second research hypothesis that the level of COVID-related stressors serves as a moderating factor in the association between lifestyle changes due to COVID-19 and psychological distress, such that among those who perceive the pandemic and the associated circumstances as more stressful, there will be a stronger association. We demonstrate the moderating effect of subjective perceptions of the situation as stressful. This finding allows us to uncover the possible mechanisms that underlie people’s psychological resilience in overwhelming events such as the COVID-19 pandemic. These mechanisms highlight the significance of subjective cognitive perceptions in this process. For example, people who reported many lifestyle changes due to the pandemic (experienced higher magnitude of change), but did not perceive the situation as stressful, demonstrated less psychological distress than people who reported fewer changes (experienced lower magnitude of change) but more COVID-19 related stress. The importance of subjective perceptions is in line with the findings of Luhmann et al. [17], whose dimensional taxonomy of perceived characteristics of major life events demonstrated that perceived event characteristics predict individual differences in changes in psychological well-being.

The present study has several strengths, including the inclusion of multiple lifestyle changes in behavioral habits, a large sample size, and the timing of data collection relative to the onset of the COVID-19 pandemic and lockdown restrictions in Israel. However, this study has several limitations resulting from its cross-sectional design. The first limitation is that, due to the cross-sectional design, causality cannot be inferred. The second limitation is that although the cross-sectional design is sufficient for collecting data during real-time crisis events, the data represent the psychological distress of the participants at the time of data collection. There is no information on the participants’ psychological distress development over time (e.g., their distress before the onset of the pandemic or changes over time). Considering that psychological distress is not static and changes over time, future studies should continue to monitor it. For example, it is possible that as the crisis continues, people’s distress will further increase (with more lifestyle changes); alternatively, some may develop resilience over time. These changes could be explored in a longitudinal study. Additionally, the current study is also limited by the use of self-report measures. The exclusion of some of the questionnaires due to a response pattern that could indicate a reliability problem (details are presented in the Methods section) may have helped to overcome this limitation. Nevertheless, this limitation should still be taken into account.

## 5. Conclusions

In conclusion, our findings indicate that changes in lifestyle behavioral habits (regardless of their nature)—positive health-enhancing behaviors and negative behaviors—are associated with increased psychological distress. Therefore, at times of overwhelming events such as the COVID-19 pandemic and its associated circumstances, it appears that people should carefully consider whether or not to make changes to lifestyle habits. Although health-enhancing behaviors, such as increasing physical activity or decreasing cigarette smoking, may be associated with physical health, these may have different association with psychological health.

Additionally, our data highlight the importance of understanding the subjective perceptions of the situation as being stressful for psychological resilience, as less stress is associated with less psychological distress, even if a person experiences many lifestyle changes. This finding suggests that a possible way to enhance psychological health among the public is through working on personal and mental perceptions of the situation. While influencing public perceptions is not easy, it is possible to implement it by adopting stress-relieving strategies.

## Figures and Tables

**Table 1:** Sample characteristics ^1^.

Demographic Measures	Descriptive Statistics ^1^
Age	40.40 (13.76), Range: 20–75
Years of education	14.62 (2.50), Range: 10–21
Gender	Female—55.10% (*n* = 1085) Male—44.90% (*n* = 884)
Income status since COVID-19	Not affected—35.24% (*n* = 694) Moderately affected—33.87% (*n* = 667) Severely affected—30.87% (*n* = 608)
Work status during COVID-19	Currently working—61.80% (*n* = 1217) Not currently working—38.19% (*n* = 752)

^1^ Continuous variables are presented as the mean (SD) and categorical data as % (*n*).

**Table 2:** Description of the study variables ^1^.

Variable	Mean	SD	Median	Range	% Above Normal
DASS Depression	4.41	5.00	3	0–21	35.96% (*n* = 705)
DASS Anxiety	2.74	4.15	1	0–21	25.76% (*n* = 505)
DASS Stress	4.87	5.01	3	0–21	25.86% (*n* = 507)
DASS Total	12.03	13.21	7	0–63	-
COVID-related stressors	32.86	8.22	33	13–52	-
Magnitude of change	3.24	1.70	3	0–10	-

^1^ A score higher than 4 indicates depression, a score higher than 3 indicates anxiety, and a score higher than 7 indicates stress.

**Table 3:** The differences in psychological distress due to change in the five lifestyle variables.

Change Variable	No Change	Positive Change	Negative Change	Welch’s *F* (df)	*p* (η^2^)
Alcohol consumption	6.43 (7.00) *n* = 349	11.53 (10.63) *n* = 166	12.85 (10.67) *n* = 157	33.97 (2, 283.73)	<0.001 (0.096)
Cigarette smoking	11.41 (12.68) *n* = 1603	11.92 (13.92) *n* = 147	16.98 (15.55) *n* = 204	12.04 (2, 260.73)	<0.001 (0.017)
Eating habits	5.26 (5.83) *n* = 406	11.09 (11.65) *n* = 812	12.64 (11.26) *n* = 643	126.15 (2, 1216.64)	<0.001 (0.065)
Sleep quality	4.21 (4.39) *n* = 463	14.05 (11.45) *n* = 972	28.98 (17.76) *n* = 111	360.85 (2, 277.03)	<0.001 (0.273)
Physical activity	8.09 (10.05) *n* = 310	10.82 (10.67) *n* = 290	12.53 (12.30) *n* = 720	18.29 (2, 669.66)	<0.001 (0.024)

## Data Availability

The data presented in this study are available on request from the corresponding author.
